# *In Silico* and *In Vitro* Comparison of HIV-1 Subtypes B and CRF02_AG Integrases Susceptibility to Integrase Strand Transfer Inhibitors

**DOI:** 10.1155/2012/548657

**Published:** 2012-07-05

**Authors:** Xiaoju Ni, Safwat Abdel-Azeim, Elodie Laine, Rohit Arora, Osamuede Osemwota, Anne-Geneviève Marcelin, Vincent Calvez, Jean-François Mouscadet, Luba Tchertanov

**Affiliations:** ^1^LBPA, CNRS, LabEx LERMIT, Ecole Normale Supérieure de Cachan, 61 Avenue du Président Wilson, 94235 Cachan, France; ^2^School of Life Science, East China Normal University, Shanghai 200062, China; ^3^Laboratoire de Virologie, AP-HP, Hôpital Pitié-Salpêtrière, EA 2387, UPMC Université Paris VI, 75013 Paris, France

## Abstract

Most antiretroviral medical treatments were developed and tested principally on HIV-1 B nonrecombinant strain, which represents less than 10% of the worldwide HIV-1-infected population. HIV-1 circulating recombinant form CRF02_AG is prevalent in West Africa and is becoming more frequent in other countries. Previous studies suggested that the HIV-1 polymorphisms might be associated to variable susceptibility to antiretrovirals. This study is pointed to compare the susceptibility to integrase (IN) inhibitors of HIV-1 subtype CRF02_AG IN respectively to HIV-1 B. Structural models of B and CRF02_AG HIV-1 INs as unbound enzymes and in complex with the DNA substrate were built by homology modeling. IN inhibitors—raltegravir (RAL), elvitegravir (ELV) and L731,988—were docked onto the models, and their binding affinity for both HIV-1 B and CRF02_AG INs was compared. CRF02_AG INs were cloned and expressed from plasma of integrase strand transfer inhibitor (INSTI)-naïve infected patients. Our *in silico* and *in vitro* studies showed that the sequence variations between the INs of CRF02_AG and B strains did not lead to any notable difference in the structural features of the enzyme and did not impact the susceptibility to the IN inhibitors. The binding modes and affinities of INSTI inhibitors to B and CRF02_AG INs were found to be similar. Although previous studies suggested that several naturally occurring variations of CRF02_AG IN might alter either IN/vDNA interactions or INSTIs binding, our study demonstrate that these variations do affect neither IN activity nor its susceptibility to INSTIs.

## 1. Introduction 

The *pol*-encoded HIV-1 integrase (IN) is a key enzyme in the replication mechanism of retroviruses. It catalyses the covalent insertion of the viral cDNA into the chromosomes of the infected cells [1]. Two reactions are required for covalent integration of viral DNA. First, IN binds to a short sequence located at either end of the long terminal repeat (LTR) of the vDNA and catalyzes an endonucleotide cleavage, 3′-processing reaction, resulting in the removal of two nucleotides from each of the 3′-ends of LTR and the delivery of hydroxy groups for nucleophilic attacks. The trimmed DNA is then used as a substrate for strand transfer (ST) reaction, leading to the covalent insertion of the DNA into the host genome [1]. Inhibitors of the strand transfer reaction—INSTIs—constitute a novel family of antiretroviral (ARV) drugs, with raltegravir (RAL) at the cape, which is a first INSTI approved for AIDS treatment. Other inhibitors in advanced phase of development are elvitegravir (ELV) and GSK572.

Human immunodeficiency virus type one (HIV-1) exhibits an exceptional level of genetic variability, which may influence the viral properties such as infectivity, transmissibility, or response to antiviral treatment [2]. The most prevalent HIV-1 group M genetic forms are subtypes A, B, C and circulating recombinant form CRF02_AG. 

Analysis of the global distribution of HIV-1 subtypes and recombinants in the two followed three-year periods, 2000–2003 and 2004–2007, indicated a broadly stable distribution of HIV-1 subtypes worldwide with a notable increase in the proportion of circulating recombinant forms (CRFs), a decrease in unique recombinant forms (URFs), and an overall increase in recombinants [3]. Particularly, in 2004–2007, CRF02_AG accounted for 8% of all global infections, following subtypes C (48%), A (12%), and B (11%). CRF02_AG is the predominant HIV strain circulating in West and West Central Africa [4–6]. Recently the recombinant CRF02_AG form was identified in the Amazon region of Brazil [7] and in China [8]. 

In France the frequency of antiretroviral-naive chronically HIV-infected patients infected with a non-B subtype reached 42% in 2006/2007, having increased significantly since 1998 (10%) and 2001 (33%). This evolution in subtype distribution was mainly due to a higher proportion of patients originating from sub-Saharan countries. Among these non-B subtypes, the most prevalent was CRF02_AG with a stable proportion around 20% between 2001 and 2006/2007 [9].

Enzymatic and virological data support the concept that naturally occurring polymorphisms in different non-B subtypes can affect the susceptibility of HIV-1 to different antiretroviral drugs, the magnitude of resistance conferred by major mutations, and the propensity to acquire some resistance mutations [10]. The genetic variation between viral isolates retroviral enzymes is estimated up to 25–35%; particularly the *pol *gene exhibits high variation, about 10–15% for reverse transcriptase (RT) and 8–12% for integrase (IN) [11]. Integrase inhibitors are active *in vivo* against B and non-B subtypes. Furthermore, *in vitro* studies suggested that subtype C integrase is equally susceptible to INSTIs [12]. Similarly, the analysis of *pol* gene in infected patients showed that highly prevalent polymorphisms have little effect on INSTIs susceptibility [13]. Nevertheless, the comparison of IN sequences of B and CRF02_AG strains showed that CRF02_AG sequence differs from the B sequence by 13 residues (K/R14, V/I31, L/I101, T/V112, T/A124, T/A125, G/N134, I/V135, K/T136, V/I201, T/S206, L/I234 and S/G283) [14]. Based on a model of the B HIV-1 integrase/DNA complex [15], it was suggested that several of these variations K/R14, T/V112, T/A125, G/N134, K/T136, and T/S206 may impact IN interaction with DNA or IN susceptibility to INSTIs. Later we compared the genetic barriers between B and CRF02_AG strains; we found that the variability between subtypes impacted the genetic barrier for G140C/S and V151I with a higher genetic barrier being calculated for subtype CRF02_AG suggesting a great difficulty in selecting these mutations for CR02_AG compared to subtype B [16]. 

**Scheme 1:**
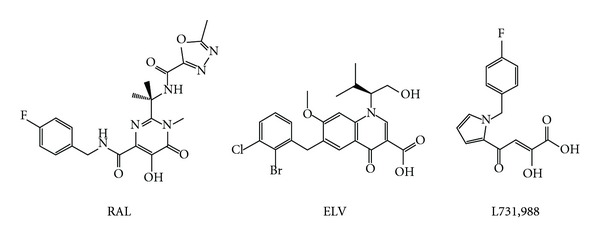


Integrase is a 288 amino acids enzyme, which consists in three structurally distinct functional domains [17]. Structures reporting HIV-1 IN single- or two-domain data allow the generation of biologically relevant models, representing either unbound dimeric enzyme or IN complexes with viral (vDNA) and/or host DNA (hDNA) [18]. The X-ray structures of full-length prototype foamy virus (PFV) IN complex with its cognate DNA and integrase strand transfer inhibitors (INSTIs; RAL, ELV, and others first- and second-generation INSTIs) were recently solved [19, 20]. The reported structures were used for homology modeling of the unbound IN and IN bound to vDNA from CRF02_AG and B strains. Further, the constructed models were used to estimate the susceptibility of both INs to strand transfer inhibitors, RAL, ELV and L731,988 (Scheme 1). Results from molecular modeling were compared to experimental data obtained with B and CRF02_AG INs which were isolated from plasma samples of HIV-1-infected patients and then cloned and expressed *in vitro*.

## 2. Results and Discussion

### 2.1. Analysis of CRF02_AG IN Sequences from INSTI-Naive Infected Patients

The complete sequence of the IN coding region of the *pol* gene was amplified and cloned from the plasma samples of CRF02_AG HIV-1-infected patients. Four IN sequences, N^1^ to N^4^, harbored several variations among the thirteen residues that were shown to be subjected to polymorphic substitutions between CRF02_AG and B HIV-1 sequences (K/R14, V/I31, L/I101, T/V112, T/A124, T/A125, G/N134, I/V135, K/T136, V/I201, T/S206, L/I234 and S/G283; Table 1) [14]. Sequence N_1_ (CRF02_AG 33CR) displayed the seven variations K14R, T112V, T125A, G134N, K136T, T206S, S283G; N_2 _CRF02_AG 49CR the five variations T112V, T125A, G134N, K136T, S283G; N_3_ (CRF02_AG 68CR) the five variations K14R, T112V, T125A, K136T, T206S; N_4_ (CRF02_AG 52CR Q148K) the two variations T125A and T206S, along with the INSTI resistance mutation Q148K. Although Q148K is involved in INSTIs resistance, the patient from whom the IN coding DNA was derived was not exposed to the INSTI-containing treatment. Thereby we presume that Q148K may be a naturally occurring amino acid substitution.

### 2.2. Structural Comparison of HIV-1 B and CRF02_AG Integrases

In order to determine the potential impact of the natural variations on the protein activity and susceptibility to INSTIs, we built models of the IN structures corresponding to the consensus B sequence and the CRF02_AG variant differing from B subtype by twelve residues. The 18-aas C-terminal end containing the S283G was omitted since the structure of this domain was not resolved by X-ray analysis and the folding of this part of protein is extremely difficult to predict in the apo state, due to its essential length and its highly solvent-exposed position. 

**Figure 1:**
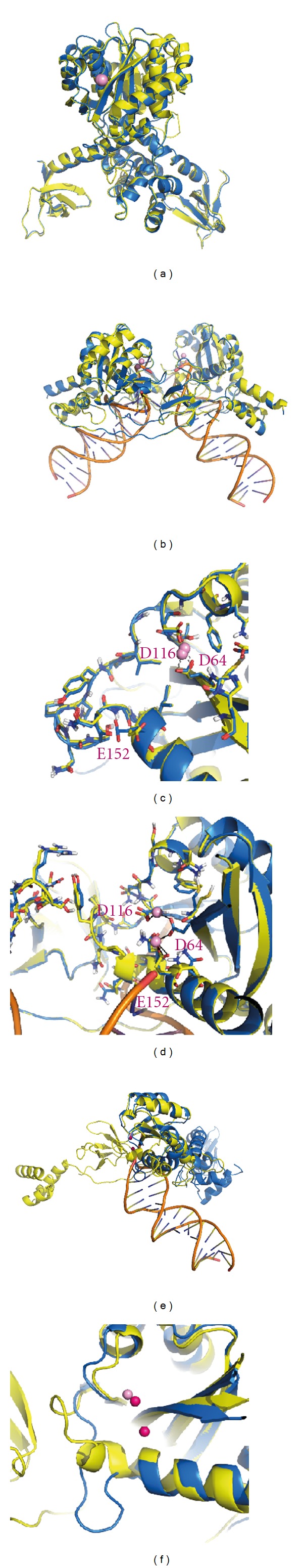
Structural models of the HIV-1 INs from B and CRF02_AG strains. (a) Superimposition of models 1 and 2, representing the enzyme before the 3′ processing from B (in blue) and CRF02_AG (in yellow) strains; (b) Superimposition of models 3 and 4, representing the IN·DNA pre-integration complex from B (in blue) and CRF02_AG (in yellow) strains; (c) and (d) Comparison of the catalytic site and loop 140–149 structure in models 1/3 (in blue) and 2/4 (in yellow) respectively. The proteins are shown as cartoons, Mg^+2^ ions as spheres (in pink). (e and f) superimposition of the structural subunits from models 1 (in blue) and 3 (in yellow) and the structural details of the active site and loop 140–149.

Comparative structural analysis were performed considering 6 IN models generated by homology modeling (Figure 1). Models 1(B) and 2 (CRF02_AG) (Figure 1(a)) represent the unbound homodimer of integrase (IN^1–270^), which depicts the conformational state of the enzyme just before the 3′-processing of vDNA (apo state); models 3′(B) and 4 (CRF02_AG) (Figure 1(b)) represent the IN dimer in complex with vDNA (holo state), which depicts the active unit of the IN·vDNA strand transfer intasome; models 5 (B) and 6 (CRF02_AG) (not shown) were derived from models 3 and 4 by removing vDNA.

Models 1 and 2 were constructed from the crystallographic structures of HIV-1 IN-isolated domains or pairs of domains. Overall, the analysis of the models representing the HIV-1 IN conformational state before 3′-processing (apo state) did not show any significant structural change between the two subtypes (Figures 1(a) and 1(c)). 

Models 3 and 4 were constructed from the crystallographic structure of the IN·vDNA complex of the PFV intasome [19, 20]. Although the sequence identity between HIV-1 and PFV INs is low (22%), the structure-based alignment of the two proteins demonstrates high conservation of key secondary structural elements and the three PFV IN domains shared with HIV-1 IN have essentially the same structure as the isolated HIV-1 domains. Moreover, the structure of the PFV intasome displays a distance between the reactive 3′ ends of vDNA that corresponds to the expected distance between the integration sites of HIV-1 IN target DNA (4 base pairs). Consequently, we are confident that the PFV IN X-ray structure represents a good template for the HIV-1 IN model generation [21]. To obtain a robust alignment, we adjusted the targets (HIV-1 INs from B and CRF02_AG subtypes) and template (PFV IN) sequences manually, considering each structural domain separately, in order to take into account the conservation of the secondary structure (see Section 4).

**Figure 2:**
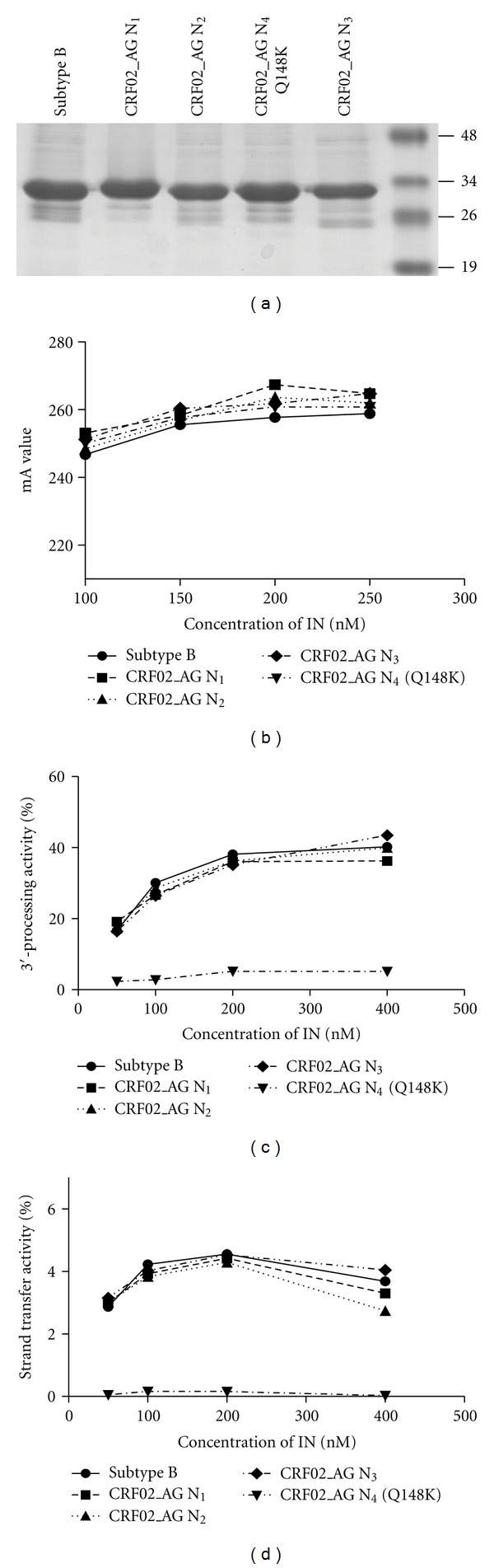
Purification of recombinant HIV-1 INs from B and CRF02_AG subtypes and comparison of their activities. (a) Purification products N_1_, N_2_, N_3_ and N_4_ of recombinant HIV-1 INs from B and CRF02_AG subtypes. (b)–(d) Comparison of DNA binding, 3′-processing and strand transfer activities, respectively, of the HIV-1 IN from B and CRF02_AG as a function of IN concentration.

 Again, models 3 and 4, representing the IN·vDNA intasomes of both strains, superimposed perfectly and no structural dissimilarity was observed (Figures 1(b) and 1(d)). Most of the variations are located far from the active sites, and the nearest two mutated residues to the active site, at positions 134 and 136, are exposed to the solvent and apparently did not affect significantly the structure. Similarly for 3′-processing, strand transfer activities of B and CRF02_AG recombinant proteins were assayed and compared. In agreement with the modeling results, activities of both INs were comparable (Figure 2(c)).

It is worth noting that large structural and conformational changes are observed between the apo (models 1 and 2) and holo (3 and 4) states regarding the relative positions of the IN domains (RMSD, root mean square deviation, of 31 Å, based on C_*α*_) (Figure 1(e)). These structural modifications result in different contacts between IN domains, N-terminal domain (NTD), catalytic core domain (CCD), and C-terminal domain (CDD). As such, in models 1 and 2 (apo state) no interaction was detected between CTD and CCD, whereas the two domains interact tightly in models 3 and 4 (holo state). The NTD-CCD interface also exhibits substantial changes: in the apo form the NTD-CCD interface belongs to the same monomer subunit whereas in the holo form the interface is from two different subunits. Moreover, IN undergoes important structural transformation leading to structural reorganization of the catalytic site loop upon vDNA binding; the coiled portion of the loop reduces from 10 residues (140–149 aas) in the apo form to 5 residues (140–144 aas) in the holo form (Figure 1(f)). This partial folding of the catalytic loop is probably stabilized through intra-IN domain-domain interactions and interactions with vDNA which contribute in the helix *α*4 elongation. 

### 2.3. *In Vitro* Enzymatic Comparison of Recombinant HIV-1 B IN and CRF02_AG IN

**Figure 3:**
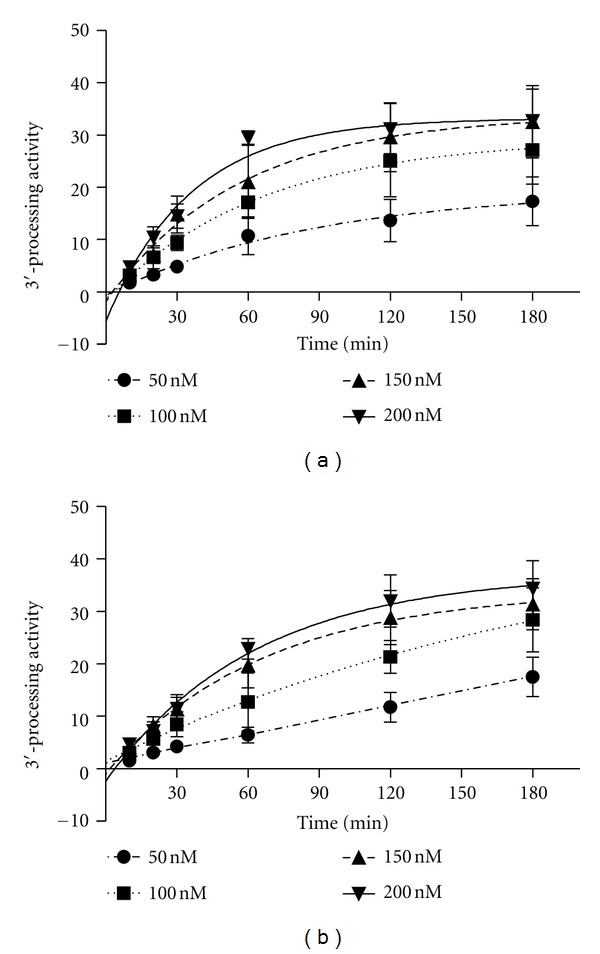
Kinetic comparison of HIV-1 B and CRF02_AG 33CR IN. (A) The kinetic features of recombinant HIV-1 B IN and (B) CRF02_AG IN (N_1_) were determined *in vitro* using 3′-processing activity assay, in the presence of 50, 100, 150, and 200 nM recombinant IN proteins with an incubation time of 10, 20, 30, 60, 90, 120, and 180 min, respectively.

To confirm experimentally the absence of divergence between INs from both strains CRF02_AG and B, N_1_ to N_4_ sequences were expressed and purified (Figure 2(a)) and their enzymatic activities were compared to the one of HxB2 B IN. First, the DNA binding activities of recombinant INs were compared using a steady-state fluorescence anisotropy assay (Figure 2(b)) [22]. In this assay, the binding of IN to a fluorophore-labeled dsODN substrate mimicking one end of the viral DNA is monitored by the increase of the steady-state anisotropy value, resulting from the restriction of the substrate movements. As shown I raln Figure 2(b), no significant difference in DNA binding activity of recombinant subtype B IN and the CRF02_AG INs was observed within a range of IN concentrations of 100 to 250 nM, thereby indicating that the variations in IN sequence did not affect the binding affinity of the enzyme. Then, 3′- processing of HIV-1 B IN and CRF02_AG INs was compared *in vitro*. No significant difference of 3′-processing activity of recombinant HIV-1 B IN and CRF02_AG INs was found within a range of IN concentrations of 50 to 400 nM (Figure 2(c)). Impaired 3′-processing and strand transfer activity, but conserved DNA binding ability of CRF02_AG 52CR Q148K were observed, in agreement with previous study [23]. Finally we decided to analyze 3′-processing kinetics of recombinant HIV-1 B IN and CRF02_AG 33CR IN in the presence of increasing concentrations of IN 50 nM to 200 nM recombinant IN proteins with an increasing incubation time, using both *in vitro* 3′-processing activity assay and steady-state fluorescence anisotropy-based assay (Figure 3). Again, no difference could be detected. This result was further confirmed by steady-state fluorescence anisotropy assay (data not shown). 

In agreement of the modeling result, *in vitro* study confirmed that the enzymatic activities of both INs were comparable.

### 2.4. Docking of INSTIs

Although B and CRF02_AG INs are structurally similar, residue variations may impact the interaction and subsequent activity of the inhibitors. To address this hypothesis, the three inhibitors RAL, ELV, and L731,988 (Scheme 1) were docked onto INs by using two different docking algorithms, Glide and AutoDock. RAL and ELV coordinates were taken from the crystallographic structures of PFV intasome cocomplexes [19, 20], L731,988 was built from scratch (see Section 4). The three compounds were considered in their deprotonated form, as it has been clearly established that diketo acids (DKAs) mainly exist in this form in solution [24]. The binding energies obtained by Glide and Autodock scoring functions are reported in Table 2.

**Figure 4:**
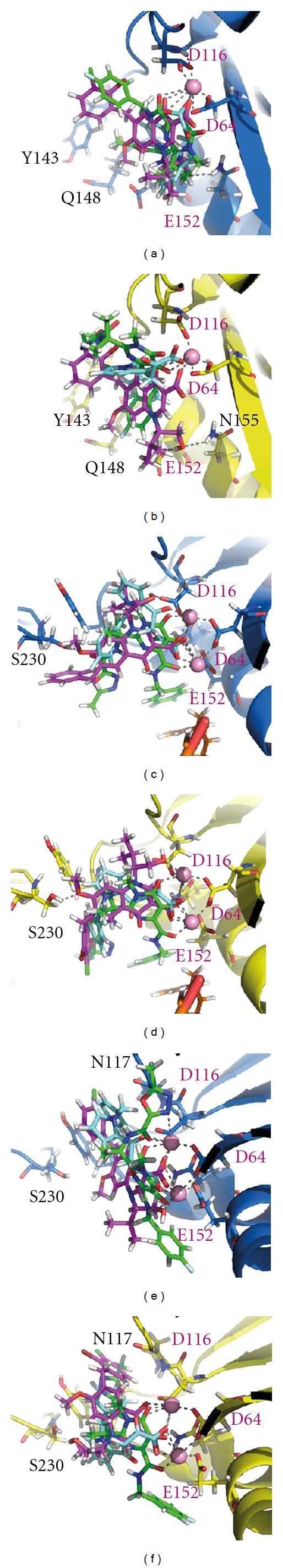
RAL (green), ELV (magenta), and L731,988 (cyan) best poses predicted by Glide. The inhibitors were docked into the active site of unbound IN (top) and IN·DNA complex (middle) and IN in holo conformation without DNA (bottom) from of the B (in blue) and CRF02_AG (in yellow). Proteins and DNA are shown as cartoons, inhibitors as sticks, and Mg^+2^ cations as balls.

The inhibitors were first docked onto the unbound IN, models 1 and 2 (apo state), with a single Mg^2+^ ion within the catalytic site. All three inhibitors are positioned at the catalytic site far from the catalytic site flexible loop. For subtype B, values of binding energies obtained with Glide range in a relatively narrow interval from −8.49 to −7.42 kcal/mol while those obtained with AutoDock range from −8.72 to −6.65 kcal/mol. Scores obtained for a given inhibitor display some variations from one strain to another and between the two docking programs. ELV best pose in model 1 (B subtype) predicted by Glide is very close to that in model 2 (CRF02_AG subtype). Small differences relate to an improved affinity of ELV to model 2 evidenced by a better score (−8.20 kcal/mol) and by the formation of an additional H-bond between the hydroxy group of ELV and E152 side chain (Figures 4(a) and 4(b)). RAL poses in models 1 and 2 differ strongly. In both cases RAL coordinates similarly the Mg^2+^ cations by its ketoenolate functionality, but the inhibitor adopts opposite positions, more specifically in model 1 its fluorobenzyl ring is oriented towards Y143, while in 2 towards Q148. L731,988 poses are also different in models 1 and 2, characterized by distinct pyrrole ring positions, close to E152 in 1 and to Y143 in 2. Such presence of alternative poses is likely due to a large pocket formed by the accessible active site and the open conformation of the folded loop which allow a large number of conformations and orientations with equivalent binding affinity for the flexible RAL and L731,988 molecules. Consequently no significant difference can be assessed between the binding of the three studied inhibitors to the unbound IN from strains B and CRF02_AG.

Further the inhibitors were docked onto models 3 and 4 representing preintegration complexes, IN·2Mg^2+^·DNA, from B and CRF02_AG subtypes, respectively. Docking resulted in a binding for the three inhibitors with significantly higher scores than those found for the apo IN. This finding agrees well with the previously published experimental data that showed a high affinity of L-731,988 only to the IN conformations adopted after assembly with the viral DNA [25]. Glide scores ranked in a range from −10.22 to −8.73 kcal/mol, while AutoDock scores range from −13.45 to −11.11 kcal/mol. Comparisons of the poses produced by the two docking software were found similar, and consequently we focus here on the analysis of Glide results. 

The three compounds are positioned in the catalytic site and chelate the Mg^2+^ cations in agreement with the mechanism of action of these molecules, which are strand transfer inhibitors [26]. RAL binding mode is characterized by higher scores in both models 3 (B subtype) and 4 (CRF02_AG subtype), respectively, to the other two inhibitors. RAL predicted poses are identical in models 3 and 4 (Figures 4(a), 4(b), 4(c) and 4(d)). It binds bidentaetly both metal cofactors of the active site acting as a 1–5, and 1–4-type ligand, with the enolic oxygen atom as an oxo-bridge between two Mg^2+^ cations. Additional stabilization of inhibitor RAL is achieved by *π*-staking of fluorobenzyl ring upon Cyt16 of DNA substrate. Similar to RAL, ELV coordinates the Mg^2+^ cofactors bidentantly through the 1–5 type *β*-ketoenolate moiety and 1–3 geminal carboxylic oxygen atoms, with a carboxylic oxygen atom as an oxo-bridge at the bicationic cluster. A few differences of ELV binding in models 3 and 4 refer to slightly different conformation of the chlorofluorobenzyl moiety. L731,988 molecule shows different binding poses in models 3 and 4. In model 3 (B subtype) L731,988 coordinates bidentately one Mg^2+^ cation by the oxygen atoms from keto functionality of ketoenolate and carboxylate groups, acting as a ligand of 1-6 type. The second Mg^2+^ cation is coordinated only by the carboxylate oxygen atom. In model 4 (CRF02_AG) L731,988 inhibitor shows exclusively one coordination to the one Mg^2+^ cation (Figures 4(e) and 4(f)). 

The predicted binding poses of RAL correlate well with those observed in the X-ray structure of the PFV intasome complex [19, 20]. Undoubtedly, the presence of the second catalytic Mg^2+^ cation, the partial loop folding, and the DNA substrate bearing are presumably the driving determinants for the tight binding of ST inhibitors in the catalytic site. It was perfectly evidenced by Cherepanov that a series of INSTIs fixed similarly to the PFV intasome [19]. Apparently the crystallographic data or static models derived from these data are not suitable means to explain the specificity of inhibitor recognition by a target. Consequently, considering the similar scoring values for a given inhibitor and closed poses, no significant dissimilarity can be assessed between the binding of studied inhibitors to the IN·2Mg^2+^·DNA complex from strains B and CRF02_AG. 

To validate the *in silico* predictions regarding the susceptibility of subtypes B and CRF02_AG INs, the efficiency of INSTIs (RAL, ELV, and L731,988) on recombinant INs proteins was determined by *in vitro* strand transfer assay in the presence of increasing concentration of INSTI (see Section 4). As to all of the three studied INSTIs, no significant difference in IC_50_ values against recombinant HIV-1 INs from B and CRF02_AG strains was observed (Table 3). IC_50_ of RAL, ELV, and L731,988 against HIV-1 INs from B and CRF02_AG strains are 41.8, 93.4, 855 nM and 13.7–25.9, 48.9–66.8, 193–291 nM, respectively. The experimental ranking of the three compounds was predicted correctly by Glide scoring function. 

The docking calculations evidenced that (i) the IN·DNA complex represents the best target for the studied inhibitors and (ii) the co-complexed vDNA partially shapes the inhibitors binding site. To further explore the role of vDNA, substrate was removed from the IN·vDNA complex and inhibitors were docked again on unbound IN with a fold corresponding to the holo state, models 5 and 6. The binding energies of RAL are depreciated upon vDNA removal in B and CR02_AG subtypes while ELV and L731,988 binding scores are less affected. 

Docking scores are nearly similar between the two strains while poses display some variations, as already observed on the apo form. Surprisingly, the AutoDock results show the lower score for RAL binding to both models 5 and 6, while the binding of the two other inhibitors are characterized by better scores, closer to those obtained with models 3 and 4. In contrast the scores produced by Glide are identical between the inhibitors and the subtypes. Chelation of the Mg^2+^ ions by the inhibitors is still maintained but the interaction patterns differ from those predicted in models 3 and 4. Indeed, in model 5 (B subtype) RAL chelates the first Mg^2+^ cation through the nitrogen atom of the oxadiazole ring, and the oxygen atom of the carboxamide moiety; the second Mg^2+^ is coordinated by 1–4 oxygen atoms of pyrimidinone fragment. In model 6 RAL mode of coordination resembles that observed in model 4; however, stabilizing *π*-stacking interactions were vanished. Again, the large volume of the binding pocket and the lack of stabilizing protein-ligand and DNA–ligand interactions can explain such variety. Consequently, unbound IN in the holo conformation, as unbound IN in the apo conformation, does not appear as a suitable target for the inhibitors RAL and ELV. L731,988 appears as a weaker binder, as confirmed by the experimental IC_50_ values.

Molecular modeling approaches were used to investigate the effect of the natural variations showed by CRF02_AG strain on the *in vitro* activities of the enzyme and its susceptibility to INSTIs as compared to the ones of the consensus B integrase. We found that the structural models of unbound (apo state) and viral DNA-bound (holo state) integrase showed very similar folding and tertiary structure for the two studied strains. The structural models of the IN·vDNA complex superimposed perfectly. This similarity was confirmed by comparable strand transfer activity for IN variants in 14, 112, 125, 134, 136, 206, and 283 positions. Consequently, the naturally occurring variations in the HIV-1 IN subtype CRF02_AG – K14R, V31I, L101I, T112V, T124A, T125A, G134N, I135V, K136T, V201I, T206S, V234I, and S283G, which were suggested to modify IN structure, do not affect significantly *in vitro* DNA binding activity, either 3′-processing or strand transfer reaction. Furthermore, docking results revealed that the modes of binding and docking conformations of three studied inhibitors are comparable for B and CRF02_AG strains and these INSTIs possessed similar IN inhibitory activity against B and CRF02_AG HIV-1 strains. Altogether these results demonstrate the absence of difference in susceptibility and confirm previously reported observations for subtype B and C HIV-1 INs [12]. Thus, in contrast to the lower baseline susceptibilities of recombinant A/G subtype virus to protease inhibitors (PIs) and reduced susceptibility of some A/G isolates to abacavir, INSTIs potentially provide an excellent therapeutic options for the treatment of HIV-1 subtype CRF02_AG-infected patients [10]. 

In the targets all three molecules are positioned similarly with keto-enol moiety in an orientation encouraging coordination of the two metal cofactors in the active site. Furthermore, independently of the method, the three INSTIs displayed a more favorable binding onto the IN·vDNA complex (holo state) than on the unbound enzyme (apo state), in good agreement with their mechanism of action [26]. Same difference in theoretically predicted modes of RAL binding was reported early by Loizidou [27]. The observed conformational and structural transformation of IN upon DNA binding led to an important change in the folding and conformation of the catalytic site loop which in turn favors a formation of the binding pocket accommodating the INSTIs. The binding modes of ELV and L731,988 were practically not altered by the removal of the viral DNA. Conversely removing vDNA had a significant effect on the docking results RAL, thereby highlighting the role of vDNA for RAL recognition most likely due to the halogenated benzyl moiety that displaces the unpaired 5′-adenine and stacking with the Cyt16 through *π*-*π* interactions. Although such interaction is thought to be involved in all the IN strand transfer inhibitors examined [19], our results suggest that ELV and L731,988 binding determinants differed in part from the ones of RAL. 

It should be noted that slight differences were observed between the results obtained with Glide and AutoDock scores, which can be ascribed to the impact of electrostatic interactions in the studied molecular systems. Indeed Glide uses higher negative charge localized on the two oxygen atoms of the hydroxypyrimidinone of RAL than AutoDock (−1.22 and −0.5*e* versus −0.183 and −0.265*e*). Also, within the AutoDock scoring function, the carboxylate charges used for ELV (2 × −0.64*e*) and L731,988 (2 × −0.62*e*) are more than two oxygen atoms attached to the pyrimidine cycle of RAL. To verify this hypothesis, we repeated the docking calculations of ELV and L731,988 using the charges of two oxygen atoms attached to the pyrimidine ring of RAL instead of those assigned by Gasteiger charges. The new binding energies of both inhibitors increased from −12.45 and −11.50 to −7.95 and −7.80 kcal/mol for ELV and L731,988, respectively. Since these atomic charges contribute highly in the binding energy as the atoms coordinate Mg^2+^ ions, they are likely responsible for the discrepancies found between the theoretical binding energies and the experimental IC_50_ values. The experimental ranking of the three inhibitors based on IC_50_ is RAL > ELV > L731,988, as predicted by Glide while the ranking predicted by the AutoDock is ELV > L731,988 ≥ RAL. The high negative charges of the carboxylate oxygen atoms of ELV and L731,988 may be the obstacle to have inhibitory actions on integrase, as efficient as RAL, since these charges increase the desolvation free energy and so increase the binding penalty for these inhibitors.

 Studies investigating the presence and frequency of polymorphisms in the HIV-1 gene of treatment-native patients are extremely important for tracing the virus evolution and the epidemiology of HIV infections worldwide. Associated crucial questions concern the effect of polymorphisms on viral enzymatic activities, susceptibility towards inhibitors, and inhibitor resistance pathways. The absence of accurate experimental data characterising the IN and/or IN·vDNA complex structures essentially perplexes an exploration of these essential topics. Since the beginning of clinical AIDS treatment with RAL in 2007, only a few attempts to probe RAL binding to integrase from different retroviral strains have been reported. Particularly, molecular docking of RAL into the IN catalytic core domain structure with the inhibitor 5CITEP as a viral DNA mimic has depicted different binding modes and affinities of RAL to IN from B and C subtypes [27]. Differences between the binding modes of several compounds to IN from B and C subtypes were also communicated [28].

In this context, our combined theoretical (structural modeling) and experimental (biochemical) evaluation of subtype CRF02_AG variation impact/effect on IN interaction with DNA or IN susceptibility to INSTIs contribute to the understanding of polymorphism effects at the molecular and structural level. Our experiments have revealed that IN from subtype CRF02_AG has similar enzymatic activity to IN from subtype B, and the susceptibility of the two INs to strand transfer inhibitors is comparable. Results from molecular modeling and inhibitor docking were found in agreement with *in vitro* observations.

Biochemical studies have revealed the impact of HIV-1 natural polymorphism on the susceptibility of protease (PR)—the other retroviral enzyme—to inhibitors [29]. Recent structural and biophysical studies have also shown that sequence polymorphisms of B and CRF01_AE strains can alter protease activity and PR inhibitors binding [30]. In this protein, the variations between the two strains directly impact the conformation of the flap hinge region and the protease core region that play crucial roles for the enzyme functions. 

By contrast, the residues showing natural variations in the HIV-1 integrases from B and CRF02_AG strains are located outside the catalytic region and outer to the binding site of the strand transfer inhibitors. Such type of polymorphism may allow the virus to preserve the integrase structural and functional properties as observed in this study. 

The methods we applied could be used for the study of other retroviral substrains emerging at the moment or to appear in the future in order to evaluate and optimize the efficiency of novel specific antiretrovirals. Consequently, our study contributes particularly to this topic and closely relates to a clinically and therapeutically—significant question—does the HIV-1 integrase polymorphisms influence the susceptibility towards integrase inhibitors?

## 3. Conclusions

The naturally occurring variations in HIV-1 subtype CRF02_AG IN, such as K14R, V31I, L101I, T112V, T124A, T125A, G134N, I135V, K136T, V201I, T206S, V234I, and S283G, do not affect notably integrase structure, neither *in vitro* enzymatic activity, 3′-processing, nor strand transfer reaction. Docking results of all the considered inhibitors into the unbound IN model show the considerably low scores respectively, to docking into the pre integration IN·DNA complex. The docking scores and inhibitor poses confirm that the generated structure of the HIV IN·DNA complex is the appropriate biologically relevant model used to explain the inhibition mechanism of the strand transfer inhibitors. All the three studied molecules are polydentate ligands able to wrap around the metal cations in the active site. The results of the docking are in perfect agreement with the proposed mechanism of action for INSTIs. Docking results reveal that the modes of binding and docking conformations of three studied molecules are identical for the HIV-1 IN from B and CRF02_AG strains. The proposed mechanism of the integrase inhibition based on considering of different conformational states, unbound IN, and IN·vDNA complex holds for the two studied strains. 

## 4. Methods

### 4.1. Molecular Modeling

 All calculations were carried out on a Linux station (4 × 2 cores) running Centos 5.4. The IN models were constructed using Modeller package 9V8 [31]. The sequence alignment was performed using ClustalW server [32, 33] (http://www.ebi.ac.uk/Tools/clustalw2/index.html). The docking of ST inhibitors, RAL, ELV and L731,988 (Scheme 1), onto the IN models 1–6 was performed using two algorithms, GLIDE [34] incorporated in the Schrödinger suite (Schrödinger Inc.) and Autodock 4.2 [35]. Figures were produced with PyMol [36]. 

### 4.2. Models of the HIV-1 IN from B and CRF02_AG Strains 

3D models of the full-length IN homodimer, IN^1–270^ (unbound IN, or *apo* state, resp. to DNA) containing one Mg^2+^ cation in each active site were generated by homology modeling from crystallographic structures of isolated pairs of IN domains. Two structures of the HIV-1 IN, one containing the N-terminal domain (NTD) and the catalytic core domain (CCD) (IN^1–210^, PDB code: 1K6Y) [37] and the other containing the CCD and the C-terminal domain (CTD) (IN^56–270^, PDB code: 1EX4) [38], were chosen as the initial templates. These structures represent multiple mutants of the HIV-1 subtype B IN, the mutations being W131D/F139D/F185K in 1K6Y and C56S/W131D/F139/F185K/C180S in 1EX4. Both structures were superimposed and CCD domain (IN^56–210^) of 1EX4, determined at lower resolution (2.8 Å) than 1K6Y (2.4 Å), was deleted. The disordered residues 271–288 were also omitted. Sequences of the WT HIV-1 INs from B and CRF02_AG strains, which differ by 13 amino acids (K/R14, V/I31, L/I101, T/V112, T/A124, T/A125, G/N134, I/V135, K/T136, V/I201, T/S206, V/I234 and S/G283), were aligned to the templates sequences using ClustalW. The missing CCD-NTD linker (47–55 aas) was constructed by an *ab initio* approach with Modeller 9V8, based on, discrete optimized protein energy (DOPE) scoring function [39]. 100 models were generated for each IN, from B and CRF02_AG strains. The conformation of the folded loop IN^140–149^ with a well-shaped hairpin structure [40] was reconstructed by a loop-generating algorithm based on database searches (Protein Loop Search). Mg^2+^ cation was inserted into the active site (D64, D116, and E152) as reported in structure 1BI4 [41] and minimized by molecular mechanics (MM) under constrains using CHARMM [42]. We shall refer to these generated models as model 1 (B strain) and model 2 (CRF02_AG strain).

### 4.3. Models of the HIV-1 IN from B and CRF0_AG Strains in Complex with vDNA 

3D models of the IN·vDNA pre integration complex (*holo* state respectively to DNA) from B and CRF02_AG strains were generated by homology modeling following a two-step procedure. The coordinates of the recently published crystal structure of the PFV IN·vDNA complex cocrystallized with RAL (PDB code: 3OYA, resolution of 2.65 $\overset{´}{Å}$) [19, 20] was used as template. The sequence alignment of the HIV-1 IN dimer (B strain) and the PFV IN was performed using ClustalW. The sequence identity between these two INs is 22%. Nevertheless, structure-based alignment of INs from the PFV and HIV-1 demonstrates high conservation of key structural elements and consequently, the PFV IN X-ray structure provides a good template for the HIV-1 IN model generation. In order to increase the quality of our model, the NED domain (residues 1 to 50), only present in PFV IN, was removed from the corresponding sequence. Then, the sequences of the structural domains of HIV-1 and PFV INs were aligned separately, taking into account the conservation of the secondary structure. The obtained sequence alignment was used for homology modeling of the HIV-1 intasome. The interdomains linker were constructed using the *ab initio* LOOP module in Modeller [43]. For both subtypes B and CRF02_AG models, distance restraints were applied to reproduce key interactions reported in earlier experimental studies [37, 44–46]. 100 models were generated for each IN, from B and CRF02_AG strains, and those with the lowest energy were retained. We shall refer to these models as model 3 (B strain) and model 4 (CRF02_AG strain). Two additional models 5 and 6 were generated by removing vDNA from models 3 and 4. 

### 4.4. Refinement of Models 1–6 and Quality Check out

 Hydrogen atoms were added by the HBUILD facility in CHARMM [42]. The resulting models were slightly minimized while constraining carbon-*α* to remove clashes. The stereochemical quality of the models was assessed with Portable ProCheck [47], which showed that more than 97% of the residues in all models had dihedral angles in the most favorable and allowed regions of the Ramachandran plot, indicating high model quality. 

### 4.5. Molecular Docking

Initial molecular geometries of ELV and RAL were taken from the X-ray structures 30YA (RAL) and 3L2U (ELV) of PFV IN·vDNA complexes [19, 20]. The 3D structure of the compound L731,988 was generated by ChemBioOffice 2010 [48]. The models of all inhibitors (Scheme 1) in deprotonated form were minimized with density functional theory (DFT) B3LYP 6-31G* method implemented in *Gaussian03* program [49]. Inhibitors RAL, ELV, and L731,988 were docked onto models 1–6 using two algorithms, GLIDE [34] and AutoDock 4.2 [35]. The receptor is considered as a rigid body while the ligand is treated fully flexible.

In AutoDock 4.2, the graphical user interface (GUI) was used for the preparation of the inhibitor and receptor files. Grid maps of interaction energies for various atom types were constructed with a grid box of dimension 25 × 25 × 25 Å^3^ centered on the active site. Calculations were performed with a population size of 150, number of energy evaluations of 5 × 10^6^, maximum number of generations of 27,000, mutation and crossover rate of 0.02 and 0.8 respectively. The number of runs was set to 100 to explore a large number of poses of the highest affinity and the Solis and Wets algorithm was used to relax the best 10% of the obtained conformations. 

In the Schrödinger suite receptor grids were generated by Glide 4.5 within an enclosing box of size 20 Å centered on the active site. Inhibitors were docked flexibly to these pre-computing grids using standard precision (SP) scoring function. For each compounds, the best-scored pose was saved and analyzed.

### 4.6. Cloning of IN Gene

 IN cDNA was derived from naïve HIV-1 subtype CRF02_AG infected patients. Plasmid pET15b- HIV-1subtype B IN (HBX2) was our lab's conservation [50]. Amplification of IN coding sequence was carried out with specific primers at 94°C for 10 min, then 28 repeat cycles (94°C for 30 s, 55°C for 45 s, and 72°C for 1 min) followed by incubation at 72°C for 10 min. PCR products corresponding to the entire IN sequences were purified and ligated into pGEM-T Easy vector (Promega) and sequenced (Eurofins MWG operon). Then IN gene was inserted into expression vector pET-15b (Novagen) after digested with Nde I and BamH I and verified by sequencing. Forward primer: 5′-CATATGTTTTTAGATGGCATAGATAAAGCC-3′;  backward  primer  for  CRF02_AG  33CR, 49CR:  5′  -GATCCTAATCCTCATCCTGTCTACCTGC-3′;  backward  primer  for  CRF02_AG 52CR  Q148K:  5′-GATCCTAATCCTCATCCTGTCCACTTGC-3′;  backward  primer  for CRF02_AG 68CR: 5′-GGATCCTAATCTTCATCCTGTCTACTTGC-3′. 

### 4.7. Expression and Purification of IN

 His-tagged INs were produced in *Escherichia coli* BL21-CodonPlus (DE3)-RIPL (Agilent) and purified under nondenaturing conditions as previously described [50, 51].

### 4.8. Steady-State Fluorescence Anisotropy-Based Assay

Steady-state fluorescence anisotropy values were recorded on a Beacon 2000 Instrument (Panvera, Madison, WI, USA), in a cell maintained at 25°C or 37°C under thermostatic control. The principle underlying the anisotropy-based assay was published elsewhere [52, 53]. DNA-binding assay was carried out at 25°C for 20 minutes in a buffer containing 10 mM HEPES pH 6.8, 1 mM dithiothreitol, and 7.5 mM magnesium chloride in the presence of 12.5 nM-double stranded DNA substrate (21-mer oligodeoxynucleotide mimicking the U5 viral DNA end, fluorescein-labeled at the 3′-terminal GT) and 100, 150, 200, and 250 nM recombinant IN, respectively. In kinetic study, steady-state fluorescence anisotropy-based 3′-processing activity assay was performed in the presence of 50, 100, 200, and 250 nM recombinant IN proteins and 12.5 nM double stranded fluorescein-labeled DNA substrate, at 37°C for 10, 20, 30, 60, 90, 120 and 180 min.

### 4.9. IN 3′-Processing and Strand Transfer Activity Assay

*In vitro* 3′-processing and strand transfer activities assays were carried out using the 21/21-mer or 21/19-mer double stranded oligodeoxynucleotides marked with [*γ*-^32^P] ATP-respectively, as previously described [51]. The duration of the assays was 3 hours, at temperature 37°C, in a buffer containing 10 mM HEPES pH 6.8, 1 mM dithiothreitol, and 7.5 mM magnesium chloride in the presence of 12.5 nM double stranded DNA substrate and 100 nM recombinant IN. The kinetic study was carried out by testing *in vitro* 3′-processing activity in the presence of 50, 100, 150, and 200 nM recombinant IN proteins, at 37°C for 10, 20, 30, 60, 90, 120 and 180 min, respectively.

### 4.10. Susceptibility to INSTIs

Susceptibility of INs to INSTI was determined by testing in vitro strand transfer activity in the presence of increasing concentration of INSTI in DMSO. Inhibition by the drug was expressed as a fractional product (percentage of the activity of the control without drug). The 50% inhibitory concentration (IC_50_), defined as the concentration of drug that results in 50% inhibition, was calculated from inhibition curves fitted to experimental data with Prism software, version 5.0 (GraphPad Software, Inc., San Diego, CA, USA).

## Figures and Tables

**Table 1:** Amino acid variations at the positions putatively affecting the susceptibility to INSTI in 4 isolated HIV-1 subtype CRF02_AG IN coding sequences.

Position	B consensus	Subtype CRF02_AG			
		N_1_ (33CR)	N_2_ (49CR)	N_4_ (52CR Q148K)	N_3_ (68CR)
14	K	R	K	K	R
112	T	V	V	R	V
125	T	A	A	A	A
134	G	N	N	G	G
136	K	T	T	K	T
206	T	S	T	S	S
283	S	G	G	S	S

Compared with HIV-1 subtype B IN, seven variations present at positions 14, 112, 125, 134, 136, 206, and 283 of CRF02_AG 33CR IN; five variations at positions 112, 125, 134, 136, and 283 of CRF02_AG 49CR IN; five variations at positions 14, 112, 125, 136, and 206 of CRF02_AG 68CR IN; CRF02_AG 52CR Q148K has two variations at positions 125 and 206, and an INSTI-resistant mutation Q148K, the R112 was not considered.

**Table 2:** Docking binding energies of RAL, ELV and L731,988 on the HIV-1 IN from B and CRF02_AG strains predicted by Autodock and Glide. The targets are the IN model with one Mg^2+^ cation in the active site (apo state, models 1 and 2) and IN·DNA model with two Mg^2+^ cations (holo state, models 3 and 4).

Target		The free binding energies (kcal/mol)	
	Inhibitor	Autodock	Glide
IN B (apo)	RAL	−6.83	−8.05
	ELV	−8.22	−7.42
	L731,988	−7.81	−8.49
IN CRF02_AG (apo)	RAL	−6.65	−7.68
	ELV	−8.72	−8.20
	L731,988	−8.31	−7.85
IN·DNA_B (holo)	RAL	−11.43	−10.22
	ELV	−12.45	−9.17
	L731,988	−11.50	−8.73
IN·DNA CRF02_AG (holo)	RAL	−11.11	−9.98
	ELV	−13.45	−9.16
	L731,988	−11.93	−8.82
IN^∗^ B (holo)	RAL	−8.29	−8.36
	ELV	−11.62	−8.92
	L731,988	−12.19	−8.96
IN^∗^ CRF02_AG (holo)	RAL	−7.98	−8.46
	ELV	−11.80	−8.93
	L731,988	−11.58	−8.82

**Table 3:** IC_50_ of 3 INSTIs against recombinant HIV-1 B IN and CRF02_AG IN.

	IC_50_ (M)		
	RAL	ELV	L731,988
Subtype B	4.185*e* − 008	9.340*e* − 008	8.554*e* − 007
CRF02_AG N_1_	1.373*e* − 008	5.562*e* − 008	2.115*e* − 007

## Abbreviations

HIV-1: Human immunodeficiency virus
vDNA: Viral DNA
hDNA: Host DNA
IN: Integrase
CRF02_AG: Circulating recombinant form
LTR: Long terminal repeat
PFV: Prototype foamy virus
NTD: N-terminal domain
CCD: Catalytic core domain
CTD: C-terminal domain
ST: Strand transfer
INSTI: Integrase strand transfer inhibitor
DKAs: Diketo acids
RAL: Raltegravir
ELV: Elvitegravir
PI: Protease inhibitors
RMSD: Root mean square deviation.
